# Structural and aggregation behavior of the human γD-crystallin mutant E107A, associated with congenital nuclear cataract

**Published:** 2010-12-17

**Authors:** Venkata Pulla Rao Vendra, Dorairajan Balasubramanian

**Affiliations:** Hyderabad Eye Research Foundation, L. V. Prasad Eye Institute, Hyderabad, India

## Abstract

**Purpose:**

To analyze the conformational features and aggregation properties of the mutant protein E107A human γD-crystallin (HGDC), associated with congenital nuclear cataract.

**Methods:**

cDNAs of wild type and E107A mutant were cloned and expressed in *BL21 (DE3) pLysS* cells and the proteins isolated and purified. The conformational properties and structural stability of the two proteins were compared using circular dichroism and fluorescence spectroscopic analysis. His-tagged cDNAs of the two proteins were transfected into HLE-3B human lens epithelial cells, and into HeLa cells and their in situ aggregation properties compared using immunofluorescence.

**Results:**

The mutant protein was found to be remarkably similar in its secondary and tertiary structural features to the wild type. Its structural stability, analyzed by guanidinium chloride-induced denaturation, was also found to be similar. Its solubility, however, was over hundred-fold less than that of the wild type, and it had the tendency to precipitate and form light scattering particles. That it had the tendency to self- aggregate was noticed by using bis-ANS and Nile Red as extrinsic fluorescent probes. Such aggregation was also seen in situ when transfected and expressed in HLE-3B and in HeLa cell lines.

**Conclusions:**

E107A HGDC is yet another example of how a point mutation in the protein does not affect its conformation and stability but leads to substantial reduction in solubility and generation of light scattering aggregate particles in vitro and in situ when introduced into cell lines.

## Introduction

Unlike age related cataract, which has multiple etiologies, congenital cataract is essentially due to mutations in genes (barring those that occur due to infections in utero). While over 34 mutations in a variety of genes (the crystallins, gap junction proteins, some enzymes) are known to be associated with congenital cataracts [1], the ones involving human (γ-crystallin) have attracted particular attention for detailed study. This is because γ-crystallins are monomeric proteins, and the structures of several of these are known both in solution and in the crystal state, thus making it possible to attempt protein-structure-based correlation with cataract formation. And among them, human γD-crystallin (HGDC) has been studied in some detail since more than a dozen mutations in this protein are associated with congenital cataract [2]. The molecular anatomy of HGDC has been studied in detail [3,4], and correlations between structural perturbations of some mutants and the resultant loss of solubility, phase separation and lens opacification have been made in some detail [5-7].

We focus our attention here on the mutation E107A of HGDC, which is reported to be associated with congenital nuclear cataract [8]. Our study suggests that this single replacement of glutamic acid in the COOH-terminal domain of this double-domain protein by the neutral apolar alanine leads to self-aggregation, forming light scattering particles both in the test tube and when transfected into cell lines. E107A is yet another mutant of HGDC where the secondary and tertiary structures are much the same as the wild type molecule, and yet change in a single residue leads to precipitation and light scattering.

## Methods

### Cloning of wild type and mutant human γD-crystallin (HGDC) constructs

Human cadaveric eye lens from a recently deceased person was collected from the Ramayamma International Eye Bank of our Institute, after due ethical and scientific approval from the Institutional review Board, and total RNA was isolated from it using Trizol reagent. The first strand was synthesized by RT–PCR using oligo-dT primer and Superscript III reverse transcriptase. HGDC cDNA was amplified from the first strand using forward primer with Nde 1 restriction site and reverse primer with Hind III restriction site. The amplified wild-type HGDC cDNA was cloned into a SmaI digested pBSK(+) vector. The recombinant clones were confirmed by PCR and restriction digestion. Wild-type cDNA was released from the pBSK(+) vector by restriction digestion with Nde1 and Hind III. The released cDNA was sub-cloned into the Nde1 and Hind III sites of the pET-21-a vector. E107A mutant clones were generated from the pET-21-a HGDC template by site directed mutagenesis using Phusion DNA polymerase. The amplification conditions were as follows: an initial denaturation step at 98 °C for 30 s, followed by 16 cycles of denaturation, annealing and extension at 98 °C (10 s), 55 °C (30 s), 72 °C (3 min), respectively, with a final extension step at 72 °C for 10 min. The PCR product was digested with Dpn 1 for 1hr and transformed into *DH5α* and the plasmids were isolated.

### Generation of His-tagged wild type and mutant HGDC constructs

His- tagged wild-type HGDC was amplified from the pET21-a-γD using Phusion DNA polymerase using forward primer having EcoR1 restriction site, and reverse primer having codons for His tag and Xho 1 restriction site and cloned into the EcoR1 and XhoI sites of the pCDNA3.1(+) vector by the above-mentioned method. His-tagged E107A HGDC clone was generated from the pCDNA3.1(+) γD template by site-directed mutagenesis using Phusion DNA polymerase and the above mentioned method. The sequences of the wild-type and mutant clones were verified by sequencing using an ABI 3130 Genetic Analyzer, with T7 forward,T7 reverse and BGH reverse primers. The primers used for cloning and sequencing are listed in Table 1.

**Table 1:** List of primers used for cloning and sequencing.

**Clone**	**Primer**	**Primer sequence**
pET21-a-γD	Forward	5′TCCCATATGGGGAAGATCACCCTCTACGAG3′
	Reverse	5′GCAAGCTTTCAGGAGAAATCTATGACTCT3′
pET21-a-γDE107A & pCDNA3.1(+)γDE107A	Forward	5′gatagagttcactgcggactgctcctgtcttcaggaccg3′
	Reverse	5′GACAGGAGCAGTCCGCAGTGAACTCTATCATCTGGCCTC3′
pCDNA3.1(+)γD	Forward	5′CGGAATTCATGGGGAAGATCACCCTC3′
	Reverse	5′CGCTCGAGTTAATGATGATGATGATGATGGGAGAAATCTATGACTCTCCTCAG3′
T7	Forward	5′TAATACGACTCACTATAGG3′
T7	Reverse	5′TATGCTAGTTATTGCTCAG3′
BGH	Reverse	5′TAGAAGGCACAGTCGAGG3′

### Overexpression of recombinant proteins

The recombinant constructs pET21-a-γD wild-type and pET21-a-γDE107A were transformed into *BL21(DE3) pLysS* cells. The cultures were grown at 37 °C in the presence of ampicillin and chloramphenicol to an absorbance value of 0.6 at 600 nm. Expression was induced by the addition of 1 mM isopropyl 1-thio-D-galactopyranoside, and the cultures were grown for an additional 4 h. Cells were pelleted down from 2 l culture by centrifugation at 5,850× g for 20 min at 4 °C and were suspended in 40 ml of Lysis Buffer (50 mM Tris HCl, pH 7.5, 100 mM KCl, 1 mM EDTA, 1mM DTT, 1mMPMSF, and 20 μg/ml aprotinin). Sonication was done for 40×30 s with 30 s intervals at 35% amplitude using a high intensity ultrasonic processor (Sonics Vibra Cell; Sonics & Materials Inc., Newton, MA).The cell lysate was centrifuged at 20,300× g at 4 °C for 20 min.

### Purification of the proteins

The supernatant was subjected to ammonium sulfate fractionation at 20%, 30%, and 60% concentrations. All the fractions were loaded onto a 15% SDS–PAGE gel and monitored for the presence of the recombinant protein. The fraction containing the recombinant protein (30%–60%) was taken and excess salt removed by dialysis using 50 mM Tris-Cl buffer pH 8.3 and chromatographed using an Q-Sepharose (Sigma Aldrich, St Louis, MO) ion-exchange column The Q-Sepharose column was equilibrated with 50 mM Tris-Cl buffer, pH 8.3, and the (30%–60%) protein fraction was loaded on the column and eluted using a salt gradient of 0–1 M KCl. The OD_280_ was noted and the fractions were checked by SDS–PAGE. The fractions containing the required protein were pooled, concentrated using an Amicon stirred ultrafiltration cell (Amicon, Beverly Hills, MA) with a 3 kDa cut off membrane and the concentrated protein was dialyzed against 2 mM MOPS. The recombinant proteins were further purified to homogeneity by gel filtration chromatography on Sephadex G-75 (Sigma Aldrich) column. The G-75 column was equilibrated with 2 mM MOPS and the protein sample was loaded on to the column and fractions were collected. The OD_280_ was noted and the fractions were checked by SDS–PAGE and the fractions containing required protein were pooled and concentrated using Amicon stirred ultrafiltration cell with a 3 kDa cut off membrane. The purity of the proteins was assessed by the appearance of a single band on SDS–PAGE. The concentration of each protein was calculated based on its molar extinction coefficient.

### Spectroscopic analysis of recombinant proteins

Intrinsic fluorescence spectra were recorded at room temperature using a fluorescence spectrophotometer (F-2500; Hitachi, Yokohama, Japan), and the spectra recorded in the range of 300 to 400 nm using an excitation wavelength of 295 nm, with 2.5 nm excitation and emission slits. The protein concentrations used were 10 μM (0.2 mg/ml) in MOPS buffer, pH 7.3. Extrinsic fluorescence spectra of proteins were recorded at room temperature using two surface hydrophobicity reporter probes, namely 4, 4’-dianilino-1,1’-binaphthyl-5,5′-disulfonate or bis-ANS, and 9-diethylamino-5H-benzo[alpha]phenoxazin-5-one or Nile Red. With bis-ANS, the spectra were recorded in the range of 400 to 600 nm, with the excitation wavelength at 390 nm with 2.5 nm excitation and emission slits. With Nile Red, the excitation was at 540 nm and the emission recorded between 565 and 700 nm, using 10 nm slits. The protein concentrations used, in each case, were 10 μM (0.2 mg/ml) in MOPS buffer, pH 7.3.

Circular dichroism (CD) spectra were recorded using a dichroigraph instrument (J-715; Jasco, Easton, MD) at room temperature. Far-ultraviolet (UV) CD spectra (250–200 nm) were recorded with 0.2-cm path length quartz cells and the near-UV CD spectra (320–250 nm) were recorded with 1 cm path length quartz cells. Three scans of each spectrum were averaged, smoothed, and baselines of the buffer alone were subtracted. The protein concentration used for determining the far-UV spectra was 10 μM (0.2 mg/ml) in MOPS buffer, pH 7.3 and for near-UV spectra, it was 50 μM (1.0 mg/ml) in MOPS buffer, pH 7.3.

### Cell culture and transfections

HLE-3B and HeLa cells were cultured in Dulbecco’s Modified Eagle Medium (Sigma Aldrich) supplemented with 10% fetal bovine serum (HyClone; Thermo-Fischer, Fischer Scientific, Mumbai, India) in a 5% humidified CO_2_ incubator at 37 °C. One day before transfection, 75,000 cells were seeded on a 22 mm coverslip in a six-well culture plate and incubated in a 5% humidified CO_2_ incubator at 37 °C. The medium was removed after 24 h and replaced with serum-free Dulbecco’s Modified Eagle Medium and transfected with the constructs using lipofectamine 2000 (Sigma) at a 1:2 ratio (1 μg vector/2 μl lipofectamine). After incubation for 5 h, the serum-free medium was replaced with Dulbecco’s Modified Eagle Medium containing 10% fetal bovine serum medium, and incubation continued up to 24 h for imaging. The constructs used for the transfections were pCDNA3.1(+)-WTγD, pCDNA3.1(+)-γDE107A, and pCDNA3.1(+).

### Immunofluorescence

Following incubation, the transfected cells were washed with PBS and fixed with absolute ice-cold methanol for 3 min. The cells were then washed thrice with PBS, blocked with 2.5% BSA for 1 h and incubated with anti-His antibody (1:200 dilution; Qiagen, Hilden, Germany) raised in mouse for two hours, washed three times with PBS, and incubated with FITC-conjugated anti-mouse antibody (1:500 dilution; Qiagen). Excess unbound antibody was removed by washing the coverslips thrice with PBS, and they were stained with propidium iodide for 2 min. Excess stain was removed by subsequent PBS washes. The coverslips with the cells were then mounted using 50% glycerol in PBS and images collected using a laser scanning confocal microscope (LSM510; Carl Zeiss, Jena, Germany). The excitation laser used was 483 nm. The emission of green fluorescence was collected using the 505 to 530 nm band-pass filter, and that of red fluorescence was collected using the 585 to 615nm band-pass filter.

## Results

**Figure 1:**
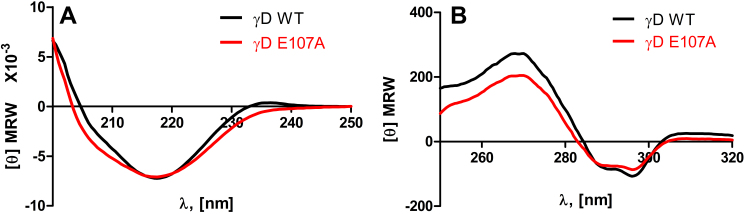
The mutation E107A in HGDC does not significantly alter the protein conformation. **A**: Far-ultraviolet circular dichroism spectra of wild-type and E107A mutant HGDC. The protein concentrations used were 10 μM (0.2 mg/ml) in MOPS buffer, pH 7.3, cell path length 2 mm, and the spectra were recorded at room temperature. [Θ]_MRW_ refers to mean residue weight ellipticity, in degrees (MRW or mean residue weight taken as 110 Da). **B**: Near UV CD spectra of wild-type and E107A mutant HGDC. Protein concentrations and the other conditions of measurement were the same as in **A**, excepting that the cells used were of a 1 cm path length.

**Figure 2:**
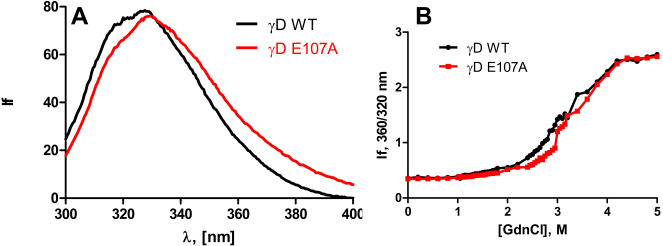
Wild type and E107A mutant differ very little in their surface exposure of apolar residues and in their structural stability. **A**: Intrinsic fluorescence of wild-type and E107A mutant HGDC. The protein concentrations used were 10 μM (0.2 mg/ml) in MOPS buffer, pH 7.3, cell path length 2 mm, and spectra were recorded at room temperature, using an excitation wavelength of 295 nm, with 2.5 nm slits. **B**: Guanidinium chloride–induced denaturation of wild-type and E107A HGDC. The relative emission intensity of the 360 nm band (of the denatured form) was compared to that of the 320 nm band (of the native protein) and monitored as a function of denaturant concentration.

Figure 1A compares the secondary structure of wild-type and E107A HGDC, using their far-UV circular dichroism spectra. The two curves are essentially superimposable, suggesting that the replacement of Glu by Ala in the molecule does not affect the backbone conformation of the protein chain in any significant manner. Figure 1B, which compares the near-UV CD spectra, reveals that the tertiary structure of the protein changes in a minor fashion (slight reduction in the residue molar ellipticity) upon the replacement of Glu by Ala. Figure 2A compares the intrinsic fluorescence spectra of the two molecules. Wild-type HGDC emits with a band maximum at 327.5 nm and intensity of 78.5 (arbitrary units), while E107A emits at 329.5 nm with an intensity of 76.2 units. Apparently, replacement of an anionic charge in the side chain of residue 107 by a nonpolar neutral moiety appears to have changed the microenvironment of the Trp and Tyr residues only in a minor manner.

We next studied the stability of the proteins by thermal denaturation. HGDC is known to be a very stable protein that does not denature even upon heating to over 75 °C [9], but starts precipitating at about that temperature [10]. Unfortunately, the mutant E107A starts precipitating at even lower temperatures (and also far sooner than the wild type, when heated up to 65 °C). Indeed, we found the solubility of the mutant to be far lower than that of the wild type, <4 mg/ml cf 400 mg/ml, respectively. We then studied the chemical denaturation patterns of the two using the denaturant guanidine hydrochloride at room temperature. Figure 2B reveals that the denaturation profiles of the two are essentially the same, as monitored by the change in the intrinsic fluorescence of the protein, from 327 nm in the native form to 350 nm upon denaturation. Both molecules show a sharp loss of conformation beyond 2.8 M of guanidine hydrochloride.

**Figure 3:**
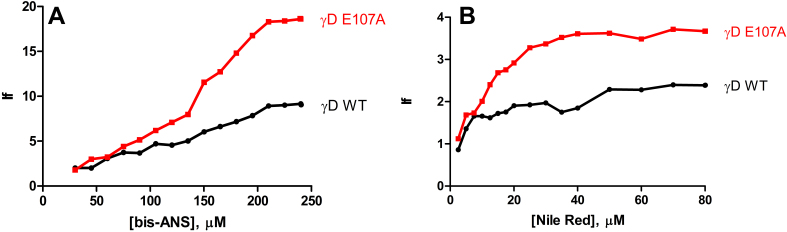
The mutant E107A protein displays remarkably higher tendency to self-aggregate than wild type HGDC. **A**: Relative emission maximum values of bis-ANS bound to wild-type and E107A HGDC, as a function of probe concentration. Excitation wavelength was 390 nm and emission 515 nm. **B**: Similar comparison of the emission maximum of the probe Nile Red bound to wild-type and E107A HGDC. Excitation maximum here was 540 nm and emission monitored at 660 nm.

In an effort to investigate the changes that might have occurred in the surface exposure of residues upon mutation, and the associated loss of aqueous solubility of the protein, we used the extrinsic reporters bis-ANS and Nile Red. Figure 3A shows that upon binding to E107A, the emission intensity of bis-ANS increases over twofold in comparison to when it is bound to the wild type, indicating the mutant to have a higher extent of surface hydrophobicity. The second probe, Nile Red, known to be a sensitive detector of protein aggregates [11,12], also showed over twofold increase in its emission intensity upon binding to the mutant (Figure 3B), in comparison with the wild type. It thus appears that the mutant has a tendency to self-aggregate and precipitate.

**Figure 4:**
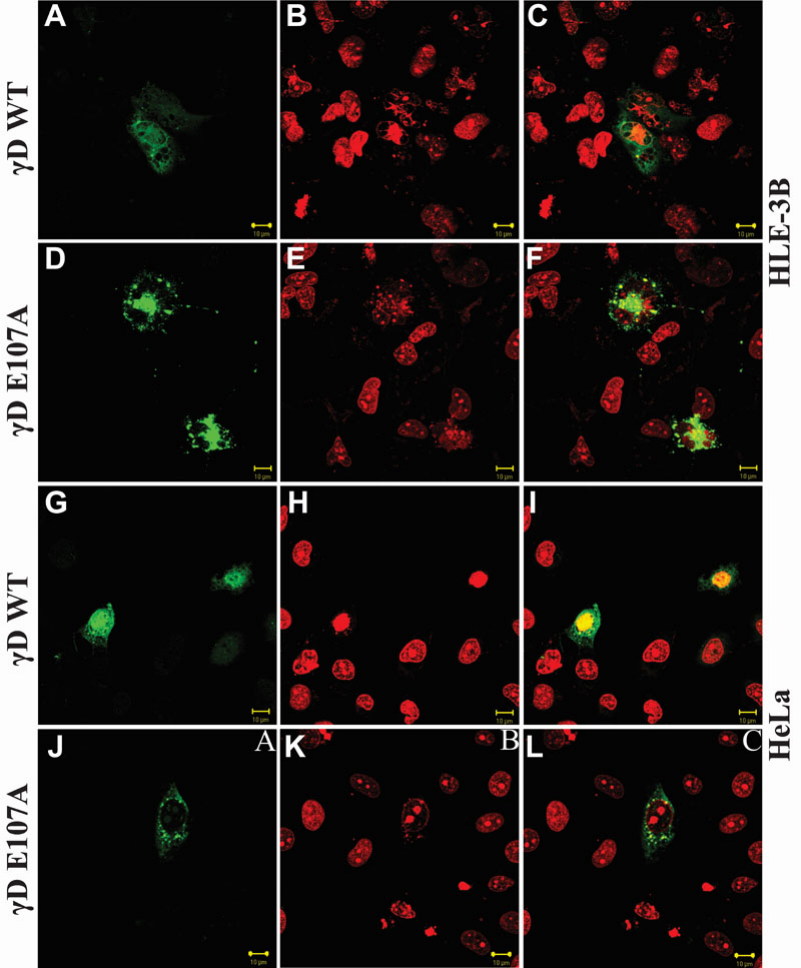
Confocal microscopic images of HLE-3B and HeLa cells transfected with His- tagged pcDNA(3.1+)γDWT and pcDNA(3.1+)γDE107A constructs. Wild-type and mutant proteins were probed with anti-his antibody (raised in mouse), and FITC conjugated anti-mouse secondary antibody. **A**, **D**, **G**, **J**: This panel set represents the protein visualized using FITC alone. **B**, **E**, **H**, **K**: This panel set is a visualization of the cells using propidium iodide as the nuclear dye. **C**, **F**, **I**, **L**: This panel set shows merged signals. Notice that the mutant shows punctate particles in both HLE-3B and HeLa cells, while the wild-type molecule does not. The signals were magnified 630×.

While the structural analysis was done on the untagged (“pure”) wild-type and mutant proteins, we have conducted the transfection experiments using His-tagged ones. This was done to differentiate the transfected protein from any native indigenously expressed HGDC in the host cell. Figure 4 shows the confocal microscopic images of human lens epithelial cells (HLE-3B cell line) transfected with the His-tagged cDNAs of wild-type and mutant HGDC, and visualized as described in the Methods section. While cells transfected with the wild-type cDNA are clear, those with the E107A mutant show light scattering particles, indicative of in situ aggregation of the mutant molecules. Quantitative analysis of the intensity of FITC fluorescence and the area of the spot covered, for both the wild-type and mutant protein spots (Figure 4A,D,G,J), using the Image J software [13], revealed the following. In the case of the wild type, the intensity was 16.04 arbitrary units and the area occupied 15,850 μm^2^, while with E107A it was 45.75 units and area 7,835 μm^2^, respectively. The mutant occupies less area, suggesting clumping and aggregation (and higher intensity from a smaller area). Similar results were obtained when we used HeLa cells (which do not express γ-crystallins [14]) rather than HLE-3B. This behavior of the E107A mutant is similar to that of W156X HGDC [15], V76D HGDC [16], and T5P γC-crystallin [17]. It was not possible for us at this point in time to determine whether these are self-aggregates or involve other proteins.

## Discussion

Replacing the anionic charged glu residue at position 107 in the COOH-terminal domain of HGDC by the nonpolar ala is expected to change the isoelectric point of the protein (from pI 7.0 of the wild type to pI 7.64 in the mutant, estimated as per [18]). Yet this change does not affect the chain conformation or the structural stability of the protein, but reduces its solubility 100-fold. In displaying this behavior, E107A is remarkably reminiscent of some other point mutants of HGDC, In particular, P23T (and its synthetic variants) was studied in considerable and comprehensive detail by the Pandes [5-7,19] and Jung et al. [20], and shows how alteration in a single side chain can have subtle but telling effects. This tendency is reflected when the protein is introduced in cell lines as well, as seen in Figure 4, with the His-tagged E107A.

While discussing the results of the in situ experiments in Figure 4, a point to be considered is whether His-tagging affects the structure and solubility of the protein. We chose to use His-tagging rather than the more common fluorescent proteins GFP, CFP, or RFP, since the latter adds more than 250 residues to the test protein (HGDC, with 178 residues), while the addition of 6 residues of His is relatively more benign. In addition, it has been shown that His-tagging has no effect on protein structure or conformation [21]. In comparison, a recent report suggests that GFP tagging of superoxide dismutase (SOD1) alters some physicochemical and fibrillation properties of the latter, though not affecting its cellular localization [22].

The mutant E107A is thus another example of the family of HGDC, besides P23T, R58H, and V76D [5-7,15], which shows how a point mutation that does not affect the secondary or tertiary structure of the protein can still generate changes in the self-aggregation behavior of the protein, ultimately leading to reduced solubility and light scattering. That it does so upon introduction in situ into cells is of some relevance to its association with cataractogenesis.
